# Resolution of Dependent Rubor after Revascularization for Peripheral Artery Disease

**DOI:** 10.31662/jmaj.2024-0237

**Published:** 2024-11-25

**Authors:** Kazuhiko Hirokawa, Hiroki Kojima, Eiji Hiraoka

**Affiliations:** 1 Department of Internal Medicine, Tokyo Bay Urayasu Ichikawa Medical Center, Urayasu, Japan

**Keywords:** peripheral artery disease, dependent rubor, dependent erythema

An 80-year-old man with hypertension, type 2 diabetes, and a 40 pack-year history of smoking presented with six months of coldness and exercise-induced pain in his left foot. In the dependent position, 5 seconds after sitting with the legs lowered, erythema was evident on the left foot (Figure 1A). In the supine position, the erythema quickly subsided (Figure 1B). This erythematous discoloration phenomenon is called dependent rubor and is a pathognomonic finding of advanced peripheral artery disease (PAD) ^(1)^. The left ankle-brachial index (ABI) was unmeasurable. Angiography revealed total occlusion of the left superficial femoral artery. Revascularization with endovascular treatment was performed. Postoperatively, the leg pain significantly improved, and the ABI improved to 0.96. One month later, the dependent rubor resolved (Figure 1C and 1D). To the best of our knowledge, the present report is the first to describe the resolution of dependent rubor after revascularization; it may indicate successful therapeutic intervention for PAD.

**Figure 1.**
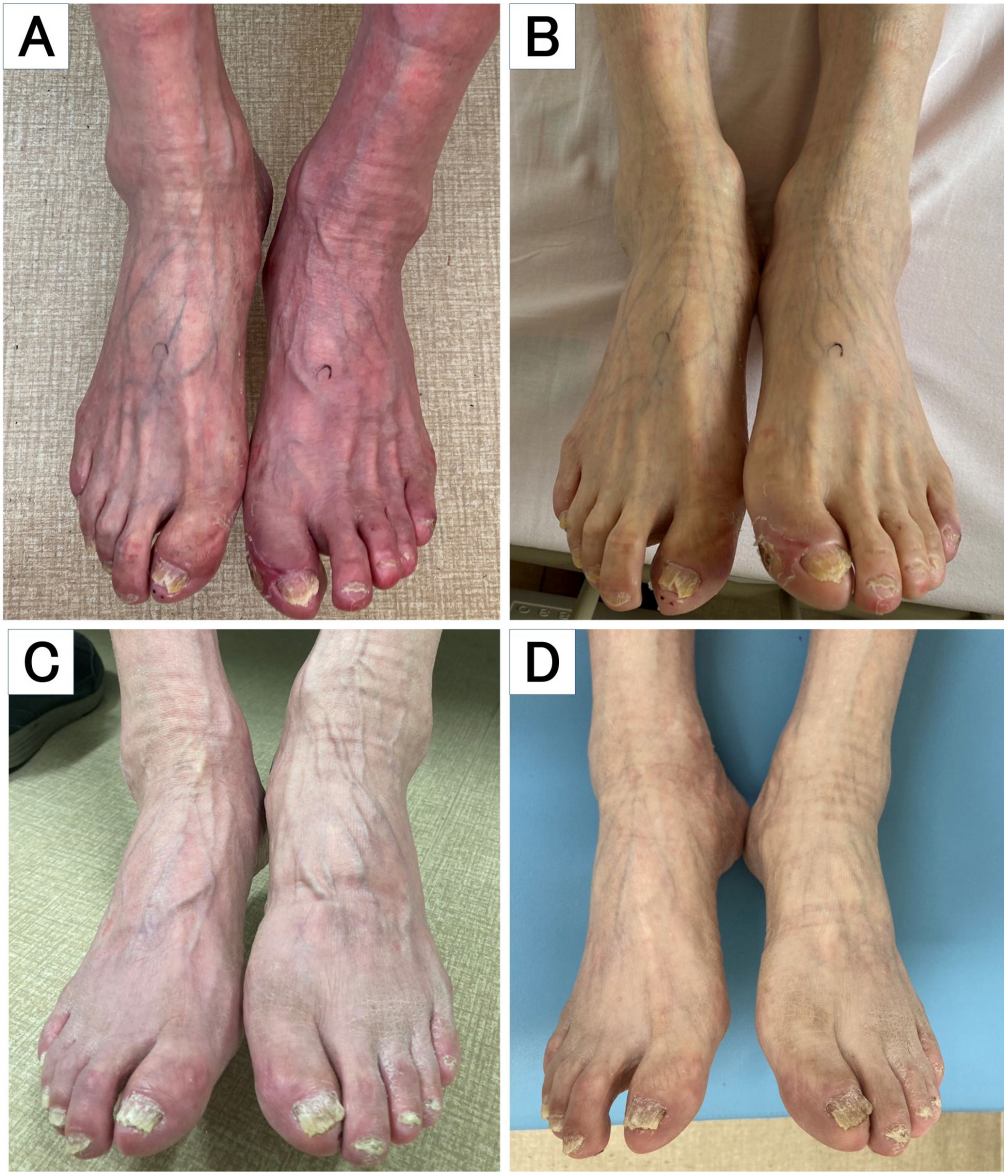
Erythematous discoloration of the left foot in the dependent position (A). Erythema disappearance in the supine position (B). Resolution of dependent rubor after revascularization in the dependent position (C) compared with the supine position (D).

## Article Information

### Conflicts of Interest

None

### Author Contributions

Kazuhiko Hirokawa: Conceptualization, Writing-original draft, Investigation

Hiroki Kojima: Writing-review & editing, Investigation

Eiji Hiraoka: Writing-review & editing

### Approval by Institutional Review Board (IRB)

This report did not require IRB approval.

### Informed Consent

Written informed consent was obtained from the patient.
